# 

**Published:** 1867-09

**Authors:** 


### Books and Pamphlets Received.

Cinical Lectures on the Principles and Practice of Medicine. By John Hughes Bennett, M. D., F. R. S. E., Professor of the Institutes of Medicine, and senior Professor of Clinical Medicine in the University of Edinburgh, etc., etc. Fifth American, from the fourth London edition, with five hundred and thirty-seven illustrations on wood. New York: Wm. Wood & Co., 61 Walker street, 1867. Buffalo: Breed, Lent & Co.

Injuries on the Eye, Orbit and Eyelids; their immediate and remote effects. By George Lawson, F. R. C. S., Eng., Assistant Surgeon to the Royal London Ophthalmic Hospital, etc., with numerous illustrations. Philadelphia: Henry C. Lea, 1867. Buffalo: Theodore Butler.

Chmistry. By Wm. Thomas Brande, D. C. S., F. R. S. L. & E., of Her Majesty's Mint, and Alfred Swaine Taylor, M. D., F. R. S., Fellow of the Royal College of Physicians of London. *Experimentis et Præceptis.* Second American edition, thoroughly revised. Philadelphia: Henry C. Lea, 1867. Buffalo: Theodore Butler.

The Physiology of Man; designed to represent the existing state of Physiological Science, as applicable to the Functions of the Human Body. By Austin Flint, jr., M. D., Professor of Physiology and Microscopy in the Bellevue Hospital College, New York, and in the Long Island Hospital College, Fellow of the New York Academy of Medicine, etc., etc. Allimentation, Digestion, Absorption. Lymph and Chyle. New York: D. Appleton & Co., 1867. Buffalo: Martin Taylor.

The Medical Use of Electricity with special reference to general Electricity as a Tonic in Neuralgia, Rheumatism, Dyspepsia, Chorea, Paralysis, and the Affections associated with General Debility, with illustrative cases. By George M. Beard, M. D., and A. D, Roxwell, M. D. New York: Wm. Wood & Co., 1867. Buffalo: Breed, Lent & Co.

Is it I? a book for every Man. A companion to Why Not? a book for every Woman. By Horatio R. Storer, M. D., of Boston, Vice President of the American Medical Association. Boston: Lee & Shepard, 1867.

Woman's Rights. By Rev. John Todd, D. D., author of Serpents in the Dove's Nest. Boston: Lee & Shepard, 1867.

Spotted or Congestive Fever. By C. B. Coventry, M. D., of Utica, N. Y.

A Plea for an Equitable Distribution of Dividends and a just settlement of Policies of Life Insurance. By Charles Cochran, M. D.

Steiger's Catalogue of German and English Books and Periodicals of Chemistry, Pharmacy, Chemical Technology, Photography, Brewing, etc. E. Steiger, importer and bookseller, 17 North William street, New York.

The Tree of Life; or Human Degeneracy. Its Nature and Remedy, as based on the elevating Principle of Orthopathy. By Isaac Jennings, M. D. New York: Miller, Wood & Co., publishers, No. 15 Laight street, 1867.

